# Non-Cancer Effects following Ionizing Irradiation Involving the Eye and Orbit

**DOI:** 10.3390/cancers14051194

**Published:** 2022-02-25

**Authors:** Juliette Thariat, Arnaud Martel, Alexandre Matet, Olivier Loria, Laurent Kodjikian, Anh-Minh Nguyen, Laurence Rosier, Joël Herault, Sacha Nahon-Estève, Thibaud Mathis

**Affiliations:** 1 Laboratoire de Physique Corpusculaire/IN2P3-CNRS UMR 6534—ARCHADE, Unicaen—Université de Normandie, 14000 Caen, France; 2 Service d’Ophtalmologie, Centre Hospitalier Universitaire de Nice, Université Côte d’Azur, 06000 Nice, France; martel.a@chu-nice.fr (A.M.); nahon-esteve.s@chu-nice.fr (S.N.-E.); 3 Laboratoire de Pathologie Clinique et Expérimentale, Biobank BB-0033-00025, Centre Hospitalier Universitaire de Nice, Université Côte d’Azur, 06000 Nice, France; 4 Service d’Oncologie Oculaire, Institut Curie, 75005 Paris, France; alexandre.matet@curie.fr; 5 Service d’Ophtalmologie, Hôpital Universitaire de la Croix-Rousse, Hospices Civils de Lyon, 69317 Lyon, France; olivier.loria@chu-lyon.fr (O.L.); laurent.kodjikian@chu-lyon.fr (L.K.); anh.minh.nguyen@chu-lyon.fr (A.-M.N.); 6 UMR-CNRS 5510 Matéis, 69100 Villeurbanne, France; 7 Centre Rétine Galien, Centre d’Exploration et de Traitement de la Rétine et de la Macula, 33000 Bordeaux, France; laurence.rosier@retinegallien.com; 8 Service de Radiothérapie, Centre Antoine Lacassagne, 06000 Nice, France; joel.herault@nice.unicancer.fr; 9 INSERM, Biology and Pathologies of Melanocytes, Team1, Equipe labellisée Ligue 2020 and Equipe labellisée ARC 2019, Centre Méditerranéen de Médecine Moléculaire, 06200 Nice, France

**Keywords:** ocular tumor, orbit, radiotherapy, brachytherapy, proton beam therapy, radiation-induced adverse events, toxicities

## Abstract

**Simple Summary:**

The irradiation of tumors involving the eye or orbit represents a complex therapeutic challenge due to the proximity between the tumor and organs that are susceptible to radiation. The challenges include tumor control, as it is often a surrogate for survival; organ (usually the eyeball) preservation; and the minimization of damage of sensitive tissues surrounding the tumor in order to preserve vision. Anticipation of the spectrum and severity of radiation-induced complications is crucial to the decision of which technique to use for a given tumor. The aim of the present review is to report the non-cancer effects that may occur following ionizing irradiation involving the eye and orbit and their specific patterns of toxicity for a given radiotherapy modality. The pros and cons of conventional and advanced forms of radiation techniques and their clinical implementation are provided with a clinical perspective.

**Abstract:**

The eye is an exemplarily challenging organ to treat when considering ocular tumors. It is at the crossroads of several major aims in oncology: tumor control, organ preservation, and functional outcomes including vision and quality of life. The proximity between the tumor and organs that are susceptible to radiation damage explain these challenges. Given a high enough dose of radiation, virtually any cancer will be destroyed with radiotherapy. Yet, the doses inevitably absorbed by normal tissues may lead to complications, the likelihood of which increases with the radiation dose and volume of normal tissues irradiated. Precision radiotherapy allows personalized decision-making algorithms based on patient and tumor characteristics by exploiting the full knowledge of the physics, radiobiology, and the modifications made to the radiotherapy equipment to adapt to the various ocular tumors. Anticipation of the spectrum and severity of radiation-induced complications is crucial to the decision of which technique to use for a given tumor. Radiation can damage the lacrimal gland, eyelashes/eyelids, cornea, lens, macula/retina, optic nerves and chiasma, each having specific dose–response characteristics. The present review is a report of non-cancer effects that may occur following ionizing irradiation involving the eye and orbit and their specific patterns of toxicity for a given radiotherapy modality.

## 1. Introduction

Ocular tumors present ophthalmologists and oncologists with rare but various clinical situations. They have in common a hierarchical therapeutic challenge that involves a multidisciplinary team and requires a sophisticated and personalized management process [1]. The challenges include tumor control, as it often influences survival; organ (usually the eyeball) preservation; and the minimization of iatrogenic damage of sensitive tissues surrounding the tumor in order to preserve vision [2,3]. These aims should contribute to maintain the patient’s quality of life and should ideally translate into lower societal costs. While surgery, by enucleation or exenteration, has historically been the standard of care for ocular tumors, it has to be associated with radiotherapy to increase tumor control probability under certain circumstances. More recently, radiotherapy has been preferred to mutilation by surgery as an eye-conserving strategy. Given a high enough dose of radiation, virtually any cancer will be destroyed with radiotherapy. Yet, the doses to normal tissues may lead to complications, the likelihood of which increases with the radiation dose and volume of normal tissue irradiated. The physics (along with their underlying radiochemistry and radiobiology) and technical aspects of radiotherapy are designed to deliver the prescribed dose in such a way as to avoid or minimize the amount of radiation delivered to nearby normal tissues while maximizing the dose delivered to the tumor. This underpins the concept of a therapeutic risk/benefit ratio that attempts to strike a balance between complications and tumor control in a given situation. At a given dose, the probabilities of complications and control can be estimated, although usually not very accurately in clinical practice, and are also dependent on the use of modern radiotherapy techniques along with improved imaging to enhance tumor targeting.

The aim of the present review was to summarize the patterns of non-cancer effects following ionizing irradiation involving the eye and orbit for a given radiotherapy modality.

## 2. Radiobiology of Ocular Tissues

### 2.1. Normal Tissue Toxicity

Normal tissue complications that arise during or after radiotherapy are the result of the killing of critical target cells crucial for structural and functional tissue integrity. The eye and orbit include substructures of various susceptibility to radiation damage, and variable impact on functional outcomes. Complications occur through complex and dynamic processes, involving oxidative stress, radiation-inducible gene expression, cellular signaling cascades, different modes of cell death (including mitotic cell death, apoptosis, senescence, etc.), and compensatory proliferative responses. For example, the hyperproliferation of fibroblasts and deposition of collagen can result in fibrosis, which can compromise wound healing. Although small, this anatomic area harbors a number of substructures of dramatically different tissue architecture and cell components, which determines their post-radiation fate.

### 2.2. Tolerance Dose

There are several important considerations in determining tolerance doses for different tissues, and these include the radiosensitivity of the different cells in a given tissue, the proliferative organization of the tissue (that determines whether the toxicity is early or late), the volume of the tissue irradiated, and the fractionation sensitivity (see below). Tolerance doses for complications in particular tissues have been assessed by pooling clinical outcome data from hundreds of patients over several years. Complication severity depends on the radiosensitivity of the target cell whose death precipitates the complication, and by the time–dose–fractionation schedule employed. Using eyelid skin as an example, an early effect of treatment might consist of dry or moist desquamation (killing of basal cells of the epidermis), whereas late effects might include fibrosis (loss of tissue parenchymal cells and some overproliferating dermal fibroblasts) or telangiectasia (damage to small blood vessels in the dermis, leading to abnormal regrowth). Eyelid avoidance may therefore be favored when possible (i.e., if the eyelid margin can also be spared), using lid retractors, during external beam radiotherapy.

While the dose is usually expressed in Gy, a corrective radiobiological factor is necessary with particle therapy to account for increased linear energy transfer (LET) and increased radiobiological effectiveness (RBE) [4]. For example, the mean RBE value of 1.1 for protons has been accepted for practical clinical purposes for years but is currently challenged due to rare unexplained toxicities and the rapid expansion of proton facilities worldwide [5]. The complications associated with the known increase in the RBE towards the distal part of the spread-out Bragg peak (SOBP) and the inherent risk associated with it have led to microdosimetric in silico or experimental approaches and have shown significant variations along the beam path [6]. Numerous clinical and translational studies are being performed to assess the impact of RBE on outcomes.

In carbon ion therapy, radiobiological models are integrated in radiotherapy planning software to account for the radiobiological efficacy of ions.

### 2.3. Volume Effects

Normal organs can be viewed as having “serial” or “parallel” structural organization, by analogy with electrical circuits [7]. A serial organ is one in which an injury at any anatomic point in the structure will produce a severe functional loss. The classic example of this is the optic chiasma; if there is major damage to it at any level, from a small dosimetric hotspot, for example, all function (vision) can be lost. Examples of parallel organs include the lacrimal glands. Radiation injury to a portion of one of these organs will in general just decrease its function by an amount related to the proportion of the organ that is destroyed. It will not produce a major functional deficit unless the irradiated volume is large, leading to dry-eye syndrome, which can further result in vision loss. Some organs behave as if they have both a serial and a parallel component. For instance, while the optic nerve has a predominantly serial behavior, clinical evidence suggests that it might also have parallel behavior [8]. Although this mixed behavior of the optic nerve is not well described, optic neuropathy may occur due to vascular occlusion [9] or neuronal degeneration.

### 2.4. Fractionation Sensitivity

Radiotherapy has rarely been delivered in single fractions. Historically, due to normal tissue sensitivity, there is a need for time to repair radiation damage, and given the lack of precise spatial/geometric irradiation to irradiate the tumor only, temporal fractionation is meant to exploit differential DNA repair capacity between normal and tumor tissues. Early- and late-responding normal tissues and tumors respond differently to fractionation patterns. Late-responding tissues are more sensitive to changes in dose per fraction, experiencing greater sparing with decreasing fraction size than their early-effect counterparts. The optic nerve can typically be damaged by high doses per fraction and is a rather late-responding tissue. The classical fractionated radiotherapy uses 1.8–2 Gy fractions 5 days a week over 4–7 weeks to deliver a total dose of 40–70 Gy. With such a regimen, the tolerance dose of the optic nerve, representing an “acceptable risk” of 5% of severe optic neuropathy and vision loss, is 54 Gy. It is important to note that ocular melanomas have been pilots in terms of use of hypofractionated radiotherapy to counteract the radioresistance of melanomas. Classical hypofractionated proton-beam therapy (PBT) or stereotactic body radiotherapy (SBRT) plans deliver about 60 Gy RBE in four fractions in uveal melanomas (UM) [2]. This is made possible due to excellent geometric targeting and implies that eye movements are well managed to ensure accurate dose delivery [2,3]. With such a regimen using doses over 6 Gy per fraction, the classical linear quadratic model does not apply. Therefore, the tolerance dose cannot be easily calculated. It would in theory be around 25 Gy RBE in four fractions. The optic disc may receive the prescribed dose of 60 Gy in parapapillary tumors [8]. In such cases, much above the “tolerance dose”, vision may be spared in many more patients than the model would predict. Therefore, normal tissue complication toxicity models, based on large databases, are warranted to better explain ocular outcomes.

Brachytherapy, PBT, and more recently, SBRT have become standard irradiation modalities for ocular tumors that exploit spatial selectivity to spare nearby normal tissues. Mini-beam radiotherapy pushes the concept of spatial fractionation further by intentionally traversing normal tissues with dose peaks and valleys, which has been shown to be protective [10]. Ultra-high dose rate FLASH radiotherapy is a disruptive form of radiotherapy that might be used in single fractions; it does not rely on the geometry of dose distribution nor on temporal fractionation to achieve differential repair efficiency between normal and tumor tissues but rather on dramatically different radiochemistry that exploits normal tissue oxygenation to reduce normal tissue effects [11,12]. Both mini-beam spatial fractionation and FLASH irradiation are under development.

## 3. Clinical Radiotherapy Concepts and Definitions of Tumor Volumes and Ocular Organs at Risk

Radiotherapy uses a common and standardized vocabulary defined internationally for the definition of the organs to be treated (tumor volume) and to be avoided (organ at risk, OAR) [13]. A succession of volumes strictly included in each other is built around the gross (visible) tumor volume (GTV): GTV < clinical tumor volume (CTV; includes gross tumor and infraclinical disease) < internal tumor volume (ITV; includes infraclinical disease and accounts for tumor movements) < planning tumor volume (PTV; accounts for physical penumbra and uncertainties around CTV +/− ITV). For tumor volumes, a radiation dose to be reached; and for OAR, a radiation dose to be constrained; is identified. Specific reports have further addressed the peculiarities of radiotherapy techniques (see below).

## 4. Description of the Different Radiotherapy Techniques Used for Ocular Tumors

Radiotherapy, in its current boundaries, can schematically be divided into external beam (tele)radiotherapy or brachytherapy. Internal radiotherapy and the use of internal vectorized alpha/beta emitting radioisotopes currently belong to the field of nuclear medicine but share some common principles with radiotherapy. Similarly, while radiotherapy is usually performed by radiation oncologists (and their staff of medical physicists and dosimetrists) in a multidisciplinary understanding of patient management with surgeons, medical oncologists (who more specifically deliver systemic treatments), radiologists, pathologists, etc., it is important to note that brachytherapy (which uses radioisotopes integrated into ocular plaques) for ocular tumors is usually placed by ocular oncology surgeons and may be prescribed and validated by a multidisciplinary team.

**Figure 1:**
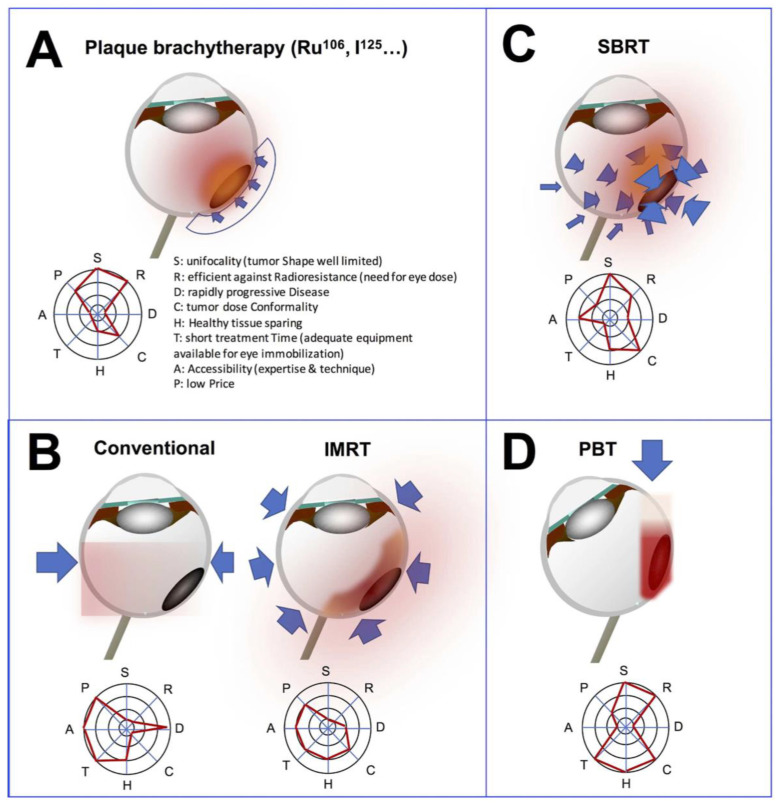
Radiotherapy techniques of and corresponding radar charts. (**A**) Brachytherapy is a conformal technique that does not deliver radiation dose outside the eye. The principle is to apply a radioactive isotope to the sclera that will deliver radiation over a short distance to the tissue. The plaque delivers a heterogeneous dose from the sclera to the apex of the tumor. (**B**) Conventional 3D radiotherapy delivers a uniform dose to the eye using 1 to 3 fields. Intensity-modulated radiotherapy (IMRT) uses 5 to 9 fields and a multileaf collimator, allowing complex concave radiation dose distribution. (**C**) Stereotactic beam radiotherapy (SBRT) delivers radiotherapy from many different positions around the organ so that the beams meet at the tumor. The tumor receives a high dose of radiation and the healthy tissues around it only a low dose. (**D**) Proton beam therapy (PBT) allows a very focused and high-dose volume of energy deposition due to the physical properties of protons. The energy is delivered with a sharp Bragg peak allowing preservation of surrounding tissues. Adapted from Mathis et al., 2019 [14].

In brachytherapy, the geometric distribution of the radiation dose is tunable by the exposure time, the geometry of the vectors inside or in contact with the tumor for the duration of brachytherapy plaque application, and the energy or the nature of emitted particles (photons, electrons). In external beam radiotherapy, Cobalt-60 (radioactive) GammaKnife^®^ radiosurgery may be used. More commonly, accelerators deliver beams of charged particles, which are either used directly (electrons, protons, heavy ions) or converted into uncharged particle beams (photons or neutrons) by the interposition of a reaction target. Linear accelerators produce electrons and photons and are typically mounted on rotating gantries and patient setup relies on a robotic couch. By placing the eye or orbit at or near the center of rotation, multiple beams can converge and overlap, delivering a high dose to the tumor area and a comparatively low dose to other areas. With heavier charged particles, directing beams is complex physically and technically (protons and ions) and restrains the versatility and affordability of the accelerators. Different types of radiotherapy techniques have been described for the treatment of ocular tumors and are detailed below (Figure 1).

Dose homogeneity has been a goal of radiotherapy plans for decades. It may, however, be less important than tumor coverage over a minimal “tumoricidal” dose, regardless of dose heterogeneities. Target dose heterogeneity by use of advanced radiotherapy techniques may be a means to achieve better tumor dose conformity and better organ sparing. One of the drawbacks of dose heterogeneity is reduced reproducibility of dose deposit and increased sensitivity to changes in conditions of irradiation. While the 2D technique may be the most uniform, this technique delivers unnecessary radiation doses to nearby structures. The dose conformity observed with intensity modulated radiotherapy (IMRT) is increased compared with that observed with the 3D technique, as is the sparing of critical uninvolved structures; however, dose homogeneity is worse with IMRT than with 3D irradiation. Similarly, SBRT has contributed to make the dose homogeneity aim quite obsolete. Another example is brachytherapy, which delivers very high doses to feeding vessels at the tumor base while the tumor apex will receive a minimally efficient tumor dose. Overall, PBT plans are currently mostly designed to achieve a homogenous dose through single-field uniform dose (as in the case for ocular PBT).

It is important to note that to achieve ocular tumor control while limiting normal tissue exposure, not only should the dose deposition be accurately defined but also delivered. Image-guided radiotherapy refers to the use of increasingly sophisticated imaging devices that are integrated into radiotherapy equipment in-room. Planned positioning images are coregistered and fused with in-room images online (i.e., immediately before irradiation) rather than offline so that displacements are calculated and applied. Bidimensional imaging systems using orthogonal X-ray images (2D) or tridimensional (3D) cone beam computed tomography (CBCT) are the most common radiotherapy in-room guidance-systems. While these systems may be used for orbital tumors, they can neither visualize ocular soft-tissue tumors in real-time nor monitor gaze axis changes. They are therefore inappropriate for ocular tumors unless patients are under general anesthesia or have their eyes closed but their gaze angle managed in a fix position, such as by retrobulbar anesthesia (which is not without risk), during each treatment fraction [2]. However, radio-opaque surrogates (tantalum clips/fiducials implanted in the tumor or its vicinity) may fulfil both the expectations of a surrogate for tumor visualization and of reproducibility of the planned gaze angle with the eyes open. It is of note that optical surface imaging devices that do not rely on ionizing radiations are being developed [15].

Image-guidance systems may be used to achieve the most accurate targeting, by repeat positioning information feedback to the radiotherapy machine, allowing what is called tracking (i.e., adaptation of beam direction to detected movements in real time). Less sophisticated options include gating where the irradiation beam is delivered if proper gaze is achieved and interrupted when not. Once a fraction is initiated, the gaze angle should be maintained identical (and eyelids remain in the same position using retractors from one fraction to another) during the whole duration of irradiation in a given session. This requires additional in-room devices, not interfering with the beams, that could image the pupil or limbus with a video camera and that instantaneous beam interruption be feasible [16]. Without gating or tracking techniques, larger margins are added around the tumor. This is, however, at the expense of several OAR, including the optic nerve, the macula, and the lens. In contrast, with brachytherapy, eye movements are intrinsically managed as the radioactive plaque is sutured to the sclera or conjunctiva and therefore moves synchronously with the eye.

## 5. Ocular Side-Effects of Radiotherapy Involving the Eye or Orbit

Radiotherapy is associated with acute and delayed side effects; the former are typically reversible, whereas the latter are usually not. Anticipation of the spectrum and severity of radiation-induced complications is crucial to the decision of which technique to use for a given tumor.

The occurrence of radiation complications depends on various parameters such as: the total dose and fractionation of radiation used—using lower doses per fraction usually allows better healthy-tissue preservation [17]; the type of radiation used—focalized radiotherapy techniques reduce collateral damages to the healthy tissues [18]; the protection used—for example, several centers use tungsten corneal shields to protect the lens when orbital irradiation is performed [19,20]; the patient’s characteristics—female sex, younger age, vascular status (hypertension, diabetes mellitus) and individual radiosensitivity (a characteristic that is not fully understood yet) have been associated with worse tolerance to radiotherapy [19]; and the location of the tumor—according to the tumor location, several ocular and orbital radiosensitive structures may be encountered [19]. For the latter, mean radiation dose thresholds have been described for these structures (Table 1); however, these limit doses have been demonstrated for standard fractionated radiotherapy and can only partially be transcribed to hypofractionated focalized radiotherapy techniques; a dose limit to the current models used to predict radiation complications is 6 Gy (or 6 Gy RBE) per fraction. More efforts are needed from radiation oncologists and ophthalmologists/ocular oncologists for a more comprehensive reporting of radiation complications, longer follow-up, and better standardized using widely internationally accepted classifications such as the common toxicity criteria (CTC, currently in its fifth version, available at https://www.eortc.be/services/doc/ctc/, accessed on 24 February 2022). A limitation of the CTC is that it is not treatment-specific; therefore, it cannot be used to address the causality of radiotherapy but rather of the multimodal treatment when radiotherapy is not exclusive. Another limitation is that the grade of severity of therapeutic is determined using vision loss. However, vision loss can itself results from many different side-effects (cataract, optic neuropathy, maculopathy, etc.). Therefore, paraclinical examinations may provide objective measurements that can be used to grade a complication and may provide additional mechanistic information. Moreover, complications may be assessed from a functional patient-driven perspective and quality-of-life questionnaires. Efforts should be undertaken by the medical community to collect complication data prospectively, to improve prediction models for better clinical decision guidance through classical modelling approaches or machine learning/artificial intelligence.

While practical thresholds have been used in conventionally fractionated radiotherapy, normal tissue complication probability (NTCP) models may provide more relevant estimates of the risks of toxicity. NTCP modes were developed for various ocular structures after hypofractionated PBT of UM in over 1000 patients [21]. Corresponding dose metrics for the optic disc, macula, retina, globe, lens, and ciliary body correlated with clinical outcome. The near-maximum dose to the macula showed the strongest correlation with VA deterioration. The near-maximum dose to the retina was the only variable with a clear impact on the risk of maculopathy, the dose to 20% of the optic disc had the largest impact on optic neuropathy, dose to 20% of cornea had the largest impact on neovascular glaucoma, and dose to 20% of the ciliary body had the largest impact on ocular hypertension. The volume of the ciliary body receiving 26 Gy was the only variable associated with the risk of cataract, and the volume of retina receiving 52 Gy was associated with the risk of retinal detachment. The optic disc-to-tumor distance was the only variable associated with dry-eye syndrome in the absence of dose volume histograms for the lacrymal gland.

### 5.1. Dry-Eye Syndrome

Ocular surface dryness is a common side effect following ocular and/or periocular radiotherapy and may involve up to 50% of patients [22,23]. Ocular dryness may be related to quantitative and/or qualitative lacrimation dysfunction [24]. Quantitative dryness is mainly related to the irradiation of the main and especially accessory lacrimal glands that should therefore be avoided in the initial radiation plan as much as possible [17,25]. Lacrimal gland toxicity is usually around 30–40 Gy, although lower doses have been used particularly in elderly patients. Qualitative dryness is related to blepharitis, which is the most common eyelid complication following irradiation. This acute radiation-induced adverse event leads to the reduction of the tear film lipid layer [19]. Delayed eyelid complications include madarosis, telangiectasia, eyelid malpositions (cicatricial ectropion or less frequently cicatricial entropion) and eyelash disorders (trichiasis or distichiasis) which accentuate dry-eye symptoms [17,18]. Keratinization of the conjunctiva and the lid margin are less frequent and result in chronic corneal abrasion. In the most advanced cases, a complete oculo-palpebral synechia may occur.

**Figure 2:**
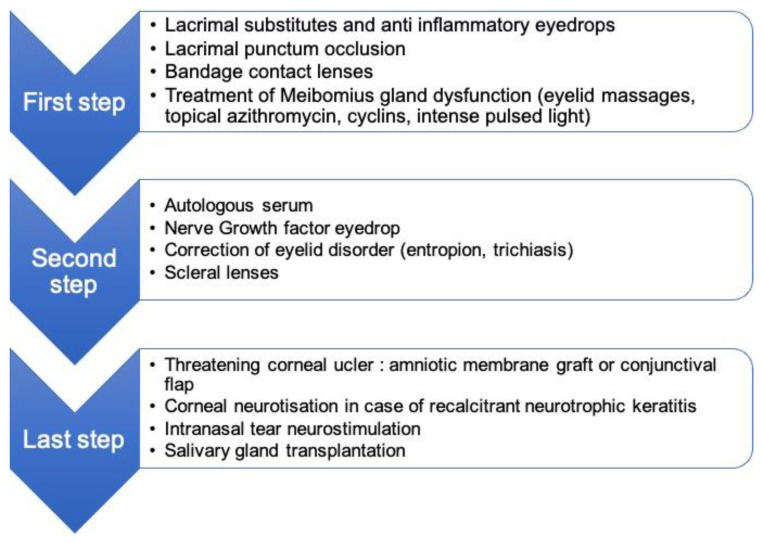
Treatment of radiation-induced dry-eye syndrome.

Severity of ocular dryness is highly variable from a patient to another, ranging from benign dry conjunctivitis to recalcitrant keratitis with eye perforation. Treatment includes topical lubricants to reconstructive surgery and should be individualized and based on the underlying clinical examination [22,26,27] (Figure 2).

### 5.2. Radiation-Induced Cataract

The lens is one of the most radiosensitive tissues in the human body and in the eye [28,29]. As such, cataract is a frequent complication of ocular or orbital radiotherapy and it occurrence ranges from 50 to 100% of patients after irradiation [30,31]. Radiation-induced cataract is often posterior subcapsular, but other subtypes can also occur. There is no pathognomonic sign or typical phenotypic presentation, which may lead to an overestimation of the prevalence, as senile or iatrogenic cataract may co-exist in a patient treated for ocular tumor. Given the limitations of the CTC and other cataract classifications, and using the Lens Opacities Classification System III (LOCS III) grading, we reported that the proton radiation dose was correlated with the rate of posterior subcapsular cataract and nuclear color cataract, diagnosed at a median 36 months post-treatment. We aimed to determine the causality of PBT, its lens-sparing potential, and its mechanisms. We found an association between the volume of the lens included in the proton field and the extent of opacities [32] and subsequently demonstrated favorable outcomes (cataract development and vision-impairing cataract) using a PBT lens-sparing approach [33]. Thus, a reduction in both the volume and dose to the lens may prevent the occurrence of cataract.

As it is easily treatable with surgery, cataracts are usually considered a minor complication of radiotherapy treatment for cancer. Previous studies have reported a significantly higher rate of postoperative complications following cataract extraction in a context of a previously irradiated globe [34,35]. Intraocular inflammation, zonular lysis, and capsular rupture leading to lens luxation seem to be more frequent after radiotherapy. In practice, cataract surgery is usually performed when there is significant visual impairment in the absence of radiation-induced optic neuropathy (RION) or maculopathy, or when the cataract hinders the fundoscopic visualization of the tumor. When vision loss is due to cataract only, and not to other retinal or optic disc complications, the overall success rate is high, and the functional outcome of surgery is very good.

### 5.3. Radiation-Induced Retinopathy

Radiation-induced retinopathy refers to an occlusive micro-angiopathy, similar to that described in diabetic retinopathy. After irradiation, endothelial cell loss and capillary closure induce hypoxic changes leading to ischemic area within the retina, and ultimately causing neovascularization [36].

The disease is characterized by telangiectasia, microaneurysms, hard exudates, hemorrhages, and edema in the irradiated retina. Aberrant retinal neovascularization represents the end stage of the disease and can be complicated by intraocular hemorrhage and tractional retinal detachment [37]. When the surface of the ischemic retina is large, neovessels can also develop on the iris margin and in the trabecular meshwork leading to neovascular glaucoma (NVG). Radiation-induced retinopathy can be associated with vision loss when macular ischemia occurs or in case of macular edema secondary to the secretion of pro-angiogenic and pro-inflammatory factors by the damaged retinal cells. Risk factors for developing radiation-induced retinopathy include the posterior localization of the tumor, a high tumor thickness and diameter at baseline, and a high radiation dose with low fractionation [38,39].

**Figure 3:**
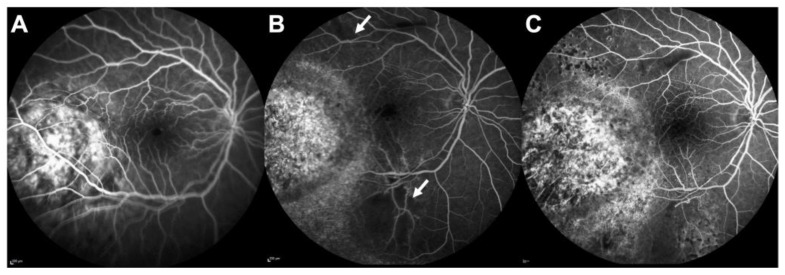
Radiation retinopathy in a patient treated with plaque brachytherapy for choroidal melanoma; (**A**) Fluorescein angiography (FA) at baseline showing the localization of the melanoma close to the macular area; (**B**) FA at 2 years showing 2 retinal ischemic areas (white arrows); (**C**) FA at 3 years showing the enlargement of the foveal avascular zone and the increased surface of ischemic areas. Laser photocoagulation was partially performed.

The time to onset after radiotherapy is approximately 18–24 months [40,41]. The incidence of radiation-induced retinopathy is highly variable between studies and can be explained by several factors: first, the methods of detection differ between studies and can influence the diagnosis of the complications. Fluorescein angiography remains the gold standard for the detection of radiation-induced retinopathy after the initial description by Hayreh [42] (Figure 3), but numerous recent studies have investigated the use of optical coherence tomography angiography (OCTA) for the detection of ischemic areas [41,43,44]. Since OCTA is a non-invasive and rapid method of examination, it can be used in routine practice for the visualization of the macular area. However, there are technical limitations to assess the peripheral retina with OCTA, and fluorescein angiography remains mandatory for whole retina evaluation [45]. The second explanation is the non-consensual grading of radiation-induced retinopathy that has evolved over time, moving from purely clinical criteria to multimodal imaging criteria [46,47,48]. Nowadays, OCT and OCTA are used in addition to widefield fluorescein angiography to provide precise grading of ischemic retinal areas and their repercussion on the macula.

The rates of radiation-induced retinopathy may be reduced by adequate use of radiotherapy. Focalized techniques, such as PBT, deliver irradiation to smaller surfaces of retina than would SBRT. The treatment of radiation-induced retinopathy is based on laser photocoagulation of ischemic retina, thus preventing the development of retinal neovascular proliferation and NVG [49]. This therapy is obviously not indicated for macular ischemic areas, as it would lead to irreversible scarring. Several treatments have been investigated for radiation-induced macular edema. Vascular endothelial growth factor (VEGF) is one of the key factors in angiogenesis, and its antagonization has shown great efficacy in the treatment of diabetic macular edema, retinal vein occlusion, and age-related macular degeneration has been extensively proven. Following these results, intravitreal anti-VEGF injections were used in radiation-induced maculopathy, aiming to decrease macular edema. Several drugs (bevacizumab, ranibizumab, and aflibercept) have demonstrated their superiority to laser photocoagulation in the treatment of radiation-induced macular edema [50,51,52,53,54]. Numerous reports have confirmed the relatively good outcomes of such treatment, with an increase in visual acuity and a decrease in macular thickness, and no increase in the reported risk of tumor recurrence or metastatic spread. Around the same period, peri-ocular and intravitreal corticosteroids have also shown favorable results in treating macular edema secondary to radiotherapy, despite the risk of steroid-induced ocular hypertension. In addition to targeting a wide range of cytokines, the main advantage of intravitreal dexamethasone (DEX) implants over anti-VEGF is their longer-lasting action, usually around 4 months, preventing frequent intravitreal reinjection [55,56,57]. To date, however, no randomized study has compared DEX implants to anti-VEGF in the treatment of radiation-induced macular edema, although it has been retrospectively shown that both treatments provide similar outcomes, with fewer intravitreal injections for patients treated by DEX implants [58,59].

A more recent approach is to prevent and reduce the occurrence of radiation-induced macular ischemia. Whenever possible, radiotherapy planning is adapted to reduce tumor margins or position a radioactive plaque eccentrically [60]. Such an approach, however, runs a higher risk of tumor recurrence [61]. Preventive measures can also be taken after irradiation, by systematic injections of anti-VEGF every 2 to 4 months. This has shown to reduce visual loss, to decrease clinical signs of radiation maculopathy, and to better preserve the foveal avascular zone on OCTA in comparison to untreated eyes [62,63,64]. It should be noted that the treatment of radiation maculopathy with intravitreal anti-VEGF is only suspensive, and that, to date, no study has evaluated outcomes after treatment discontinuation. Overall, local preventive treatment could be an interesting option in tumors at high risk of radiation maculopathy, such as large and/or paramacular tumors. However, the duration of the preventive treatment, as well as the interval between injections, still needs to be determined.

### 5.4. Radiation-Induced Optic Neuropathy (RION)

The pathogenesis of RION remains uncertain and probably occurs as a result of optic nerve radiation-related vasculopathy and neuroglial cell degeneration [65,66,67]. On the one hand, dose-dependent endothelial cell loss has been reported in the central nervous system of animals following irradiation [68] and has also been observed in the optic nerve in humans [69]; moreover, vascular inflammation, hyalinization, and vessel wall fibrosis may lead to obliterative endarteritis causing infarction and areas of necrosis in the optic nerve. On the other hand, ionizing radiation also induces somatic mutations in glial cells, leading to demyelination and neuronal degeneration [67,70].

Early clinical manifestations of RION include optic disc swelling, peri-papillary hemorrhage, dry exudates, and cotton wool spots. The majority of patients with RION develop symptoms within 3 years of treatment, with a peak at 18 months [71]. Patients usually complain of visual loss ranging from partial visual field defects to complete loss of vision according to the specific nerve fibers damaged [67]. The diagnosis is helped by fluorescein angiography, which allows the detection of optic disc leakage, and by OCT, which allows the detection of an increase in retinal nerve fiber layer thickness [72]; more recently, it has been reported that OCTA allows the identification of a decrease in radial peripapillary capillary density, and that this is correlated to the radiation dose received by the optic disc [73], which could also be of value.

The functional outcome of RION is poor. According to Kim et al., a visual acuity of ≥20/200 is retained in 52% of patients 1 year following RION, in 29% of patients after 3 years, and in 23% after 5 years. Interestingly, recovery from RION seems possible, as a spontaneous increase in vision was observed in 31% of patients over a mean follow-up of 5 years [74].

**Figure 4:**
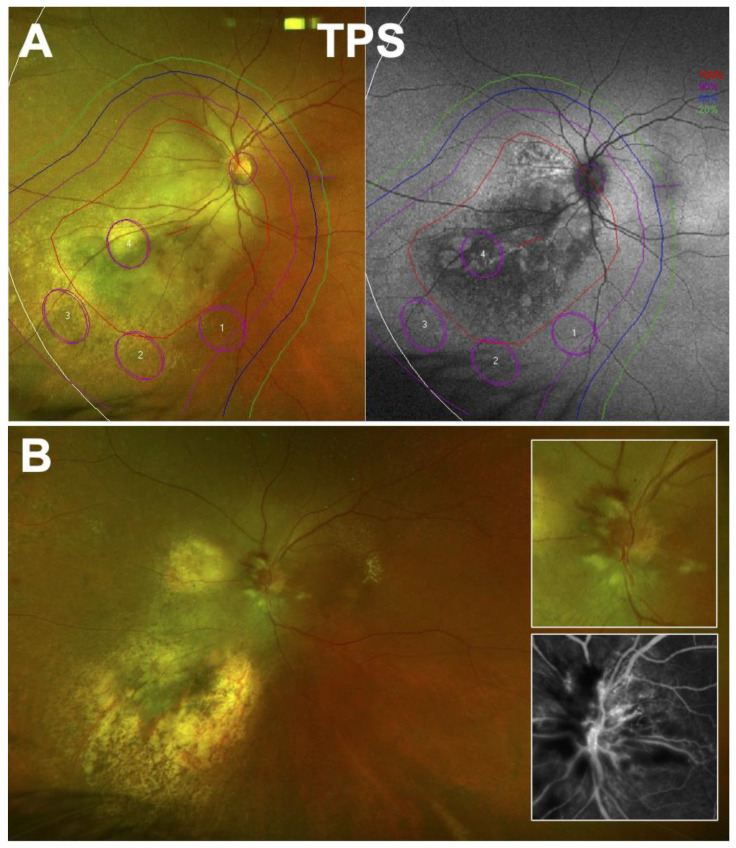
Radiation-induced optic neuritis following proton beam irradiation of a juxtapapillary choroidal melanoma. (**A**) Treatment planning system (TPS) at baseline showing isodoses on fundus autofluorescence and color widefield retinography. Approximately 50% of the optic nerve was planned to receive the full radiation dose. (**B**) At 32 months after irradiation. Observation of optic disc swelling, hemorrhages and cotton wool spots; the tumor site is atrophic. Inset: enlarged view and fluorescein angiography confirming optic nerve edema.

The main risk factor for developing RION is the localization of the tumor near the optic nerve, increasing the surface and the dose given to optic fibers (Figure 4) [75,76]. Other risk factors include younger age, male sex [77,78], and diabetes [79]. The use of concurrent chemotherapy (infrequent in the management of ocular tumors) is also associated with a higher risk of RION, although chemotherapeutic drugs may themselves be associated with optic nerve toxicity [67,80]. Obviously, the total radiation dose to the optic nerve as well as the fractionation play a role in the development of this complication [81,82].

To date, there is no proven effective treatment of RION. A few small case series reported improvement in vision after hyperbaric oxygen therapy, only if this is administered a few days after the onset of symptoms [83]. More recently, case reports and uncontrolled studies have reported rapid resolution of optic disc edema accompanied, in some cases, by visual improvement in patients treated with intravitreal corticosteroids or anti-VEGF injections [84,85,86]. However, the small numbers of patients, the lack of controls, and the short follow-up of these studies preclude any conclusion on the efficacy of local intravitreal treatment, and some authors have found contradicting results [87]. Perhaps the best approach, similarly to radiation-induced retinopathy, is to prevent the occurrence of RION. For this, the radiation dose given to the optic disc, which is the main risk factor, may be reduced by adjusting tumor margins. Moreover, reducing the length of the optic nerve irradiated, as performed by PBT, may also further reduce the risk of RION [8]. These sophisticated customizations of radiotherapy are hardly feasible with other radiotherapy techniques than PBT. In addition, systematic injections of anti-VEGF every 2 months [62] or every 4 months [63] has been proposed, leading to encouraging short-term results with respect to RION occurrence. Despite these recent modifications, RION remains a serious complication with limited therapeutic options and poor functional outcomes.

### 5.5. Toxic Tumor Syndrome

Toxic tumor syndrome refers to the ischemic and exudative retinal detachment following the irradiation of large tumors. The release of inflammatory cytokines by the necrotizing tumor, in addition to VEGF secreted by the irradiated retina, can lead to exudative retinal detachment or aggravate a pre-existing retinal detachment. The ischemic detached retina and the ischemic tumor both favor angiogenesis that can lead to rubeosis iridis and NVG. The surface of retina irradiated may be reduced using adequate radiotherapy techniques and planning. In the case of large tumors, for example, the addition of a wedge filter may slightly spare part of the retinal from irradiation [88]. The association of those three signs (retinal detachment, rubeosis iridis, and NVG) was first termed “toxic tumor syndrome” by Damato [89,90].

According to the intensity of the syndrome, and the localization and the size of the tumor mass, several treatments can be proposed. Intraocular corticosteroids and anti-VEGF have proved to be effective in treating macular edema and exudative retinal detachment, although repeated injections may be needed [91,92,93]. In such cases, DEX implants could be a valuable option [94], with a wider action on intraocular inflammatory mediators and a lower treatment burden [95,96,97]. Other therapeutic options, such as PDT or transpupillary thermotherapy [98], have been proposed but are no longer used.

Lastly, tumor excision may result in the resolution of exudative retinal detachment by removing the source of toxic inflammatory mediators. It can be performed by transscleral resection or by endoresection. Both techniques have been described many years ago and lead to a good outcome when performed by a skilled surgeon [89]. Tumor endoresection is a relatively safe surgical technique, but transscleral resection requires an experienced anesthetist to deeply lower the patient’s blood pressure in order to avoid intraocular bleeding during surgery. The technical difficulties and concerns of tumor cell dissemination associated with surgical manipulation of the tumor explain the limited use of these techniques worldwide. Some centers, however, have reported high rates of eye-retention and vision-sparing with no increase in the risk of local tumor recurrence and metastasis [90,99,100]. However, in some cases, tumor excision can lead to phthisis bulbi and secondary enucleation.

### 5.6. Intraocular Hemorrhage

**Figure 5:**
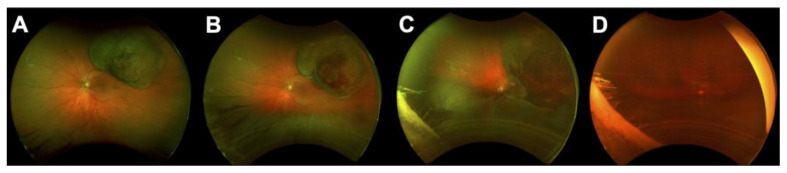
Intraocular hemorrhage following proton beam therapy for choroidal melanoma; (**A**) Retinography at baseline before irradiation. (**B**) Retinography at 6 months after irradiation showing intratumoral bleeding and toxic tumor syndrome (inferior exudative retinal detachment). The patient refused any medical or surgical intervention. (**C**) Retinography at 9 months after irradiation showing subretinal bleeding and exudative retinal detachment. (**D**) Retinography at 12 months after irradiation showing total intravitreal bleeding.

Following irradiation of a tumor, immature vessels on the surface or within the lesion are prone to intraocular hemorrhage. When the hemorrhage is intravitreal, it is easily manageable with wash-out vitrectomy, and the prognosis is usually good. However, when the hemorrhage is subretinal or choroidal, management is more complicated, and the visual prognosis is usually worse (Figure 5). It should be noted that SBRT series report higher rates of vitreous hemorrhage than PBT or brachytherapy series [101]. In contrast to PBT and brachytherapy, SBRT delivers the dose within the tumor very heterogeneously, with “hotspots” of dose that may reach 120–150% of the prescribed dose. A high dose to the vessels might be responsible for this unevenly distributed event among radiotherapy techniques.

### 5.7. Neovascular Glaucoma (NVG)

NVG is defined as the proliferation of fibrovascular membranes in the anterior chamber angle, secondary to retinal ischemia. It is a major complication of conservative treatments, as it is by far the leading cause of secondary enucleation following ocular radiotherapy [102,103]. The time to onset is about 18–24 months, and it typically occurs within the first 3 years after treatment [104]. The pathophysiology behind NVG always involves retinal hypoxia and ischemia that occurs in two situations following radiotherapy: (i) radiation-induced retinopathy responsible for retinal ischemia [36]; (ii) toxic tumor syndrome leading to exudative retinal detachment, which aggravates retinal ischemia [90].

**Figure 6:**
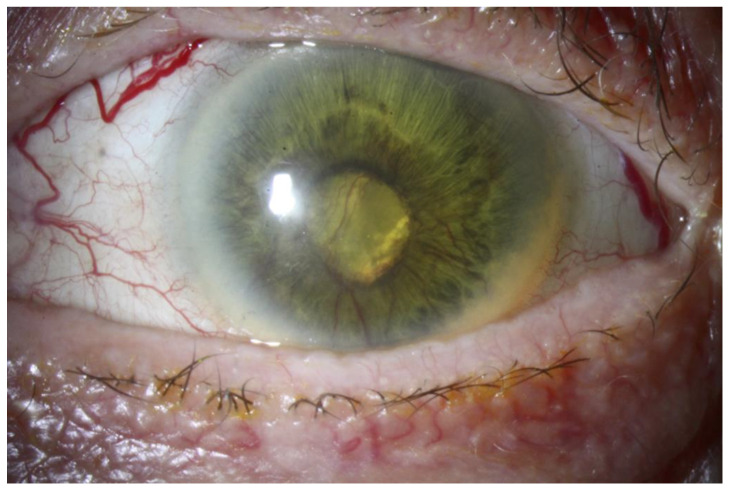
Neovascular glaucoma in a patient treated with proton beam therapy for a large choroidal melanoma (12 mm in height and 20 mm in diameter).

The key pro-angiogenic factor involved in NVG is VEGF that is secreted by hypoxic or ischemic retina, or by the tumor itself [105]. High concentrations of VEGF have been found in the aqueous humor, vitreous, and in ocular tissues of eyes with UM, and this concentration increases after irradiation [106,107,108], promoting the development of abnormal neovascular proliferation on the iris and particularly over the trabecular meshwork and leading to a rapid increase in intra-ocular pressure (Figure 6).

Higher radiation doses to the anterior chamber are one of the risk factors for developing NVG after charged particle therapy [104,109,110]. One could thus hypothesize that plaque brachytherapy for the treatment of posterior tumors should induce less NVG by sparring the anterior segment; this was confirmed Char et al., who reported fewer anterior complications, including NVG, after irradiation with ^125^I brachytherapy in comparison to helium ion therapy. However, enucleation was more frequent in the brachytherapy group due to a higher rate of local relapse [111]. Improvements in external beam radiotherapy techniques, sparing the anterior segment as much as possible, have been shown to decrease the occurrence of NVG with a higher rate of eye retention [103,104,109].

In addition to anterior segment irradiation, other risk factors for NVG have been identified such as tumor height and diameter, associated serous retinal detachment, and closer proximity to the posterior pole structure and especially the optic disc [102,103,104,112].

Laser photocoagulation of ischemic retina is the recommended treatment for radiation-induced retinopathy and leads to the regression of preretinal neovascularization in the majority of patients with proliferative disease. The regression of the iris and trabecular neovessels after panretinal photocoagulation is uncertain, maybe due to more complex pathophysiological mechanisms [113]. However, panretinal photocoagulation is helpful to prevent the development of NVG in case of ischemic retinal areas [114].

Once NVG is present, managing ocular hypertension is the most important aspect of the treatment to avoid a painful eye and further damage to the optic nerve. Topical pressure-lowering medications are usually prescribed initially, and if necessary, in association with oral acetazolamide. When this is insufficient, surgical treatment is considered, usually involving trabeculectomy with adjunctive topical anti-mitotic drugs. In recent years, glaucoma drainage implants have gained popularity as their success is less dependent on local inflammation and filtering bleb failures [115]. Non-invasive cyclodestructive procedures are also an option, although the dosage is challenging and can be insufficient, or conversely, lead to hypotony. Moreover, cyclodestructive surgery is associated with several complications including intraocular inflammation and hyphema [116,117].

Since the advent of intravitreal injection of anti-VEGF, significant improvements have been made in the management and treatment of NVG. The goal of anti-VEGF is either to stop the development of NVG or eliminate pain to avoid enucleation (30% of patients with NVG require enucleation). Anti-VEGF, administered either by intravitreal [118,119] or intracameral [120] injection, induces a regression of iris neovessels, but the vascular architecture remains, explaining the recurrence if the cause of NVG is not treated. Acting early allows the regression of iris neovessels before they invade the trabecula meshwork, avoiding an intra-ocular pressure (IOP) increase and the constitution of a fibrous neovascular membrane over the trabeculum. When angular neovessels are present but the trabecular meshwork is still open, anti-VEGF leads to a decrease in IOP [118,121]. In later stages, the effect on IOP is lower, but it has been shown that anti-VEGF decreases the risk of hyphema after a filtering surgery and is associated with a better rate of surgical success [122,123]. Thus, the early detection of iris rubeosis is essential and should prompt intravitreal injection of anti-VEGF (or intracameral if total exudative retinal detachment is present).

Overall, the management of ocular hypertension in case of NVG remains complicated and leads to enucleation in a significant number of eyes. The best way to prevent eye loss is to rapidly identify iris neovascularization before the trabeculum is invaded. This implies careful assessment of the iris margin to detect early signs of iris neovascularization, and, if present, should lead to perform rapidly anti-VEGF injections and retinal photocoagulation of ischemic areas.

## 6. Conclusions

Choosing a treatment that involves the eye and orbit implies the long-term conservation of anatomical structure and retaining a useful vision. Focalized radiotherapy techniques ensure a better control of dose delivery within the tumor, decreasing unnecessary irradiation of the surrounding healthy tissues and reducing radiation-induced vision loss. For this, better imaging systems, such as OCT technologies, choroidal doppler holography, and high-resolution magnetic resonance imaging (MRI), might increase the accuracy of the detection of tumor margins and ensure a precise delineation of the OAR [14,124,125,126]. Another way to retain visual function is preventive personalized treatment. For instance, in relatively small series, several authors have shown that the use of adjuvant treatment can reduce and delay the development of radiation-induced adverse events including radiation retinopathy, RION, and toxic tumor syndrome. Since the advent of minimally invasive intravitreal injections, anti-VEGF and DEX implants have been used and found to be effective for the treatment of macular edema and toxic tumor syndrome following irradiation. Moreover, for selected tumors and patients, the preventive use of these treatments could reduce the vision impairment at the price of injections every 2 or 3 months [64,127]. An ongoing study aimed to evaluate this strategy for high-volume melanoma (PROTECT, NCT03172299). New molecules used in age-related macular degeneration and diabetic retinopathy may also be of value for the treatment and prevention of radiation-induced retinopathy or RION. Overall, the customization of radiotherapy is largely feasible with PBT using a dedicated eyeline or with brachytherapy using binucleid notched plaques in expert centers. Although SBRT techniques are widely available, the degree of optimization of the dose distribution and customization are limited. FLASH radiotherapy may also be an area of development for normal tissue protection.

The choice of the optimal multimodal strategy and of the most appropriate radiotherapy technique, as well as its customization to a given patient with a given tumor, has been shown to be critical to eye preservation, functional, and cosmetic outcomes.

## Figures and Tables

**Table 1:** Summary of the radiation toxicity of several critical intraorbital structures.

Orbital Structure	Dose Threshold (Gy)	Toxicity	Prevention	Treatment
Lacrimal gland	30–40	Dry-eye syndrome Lacrimal duct stenosis	Delineation of the lacrimal gland during TPS	Topical lubrication Punctal occlusion
Eyelashes/Eyelid	30	Dermatitis Madarosis Eyelid malposition Trichiasis Wound healing delay	Ballistic optimization	Eyelash depilation Eyelids care
Cornea	30–40	Keratitis, Edema Stromal ulceration	Topical lubrication	Topical lubrication Topical steroids and immunosuppressive drops Bandage contact lens Lateral tarsorrhaphy Corneal graft
Lens	0.5–5	Cataract	Lens-sparing techniques	Cataract surgery
Macula	45	Ischemic maculopathy Macular oedema	Reduced margins during TPS Anti-VEGF	Anti-VEGF injections DEX-implant injections Laser photocoagulation
Optic nerve	55	Optic neuritis Optic atrophy	Reduced margins	

TPS: treatment planning system; VEGF: vascular endothelial growth factor; DEX: dexamethasone.
